# In silico screening and experimental analysis of family GH11 xylanases for applications under conditions of alkaline pH and high temperature

**DOI:** 10.1186/s13068-020-01842-5

**Published:** 2020-12-07

**Authors:** David Talens-Perales, Paloma Sánchez-Torres, Julia Marín-Navarro, Julio Polaina

**Affiliations:** 1 Department of Food Biotechnology. Institute of Agrochemistry and Food Technology, Spanish National Research Council (IATA-CSIC), Paterna, Valencia Spain; 2 Department of Biochemistry and Molecular Biology, University of Valencia, Valencia, Spain

**Keywords:** Carbohydrate-binding domain, Glycoside hydrolase, Rice straw, Xylose, Xylooligosaccharides

## Abstract

**Background:**

Xylanases are one of the most extensively used enzymes for biomass digestion. However, in many instances, their use is limited by poor performance under the conditions of pH and temperature required by the industry. Therefore, the search for xylanases able to function efficiently at alkaline pH and high temperature is an important objective for different processes that use lignocellulosic substrates, such as the production of paper pulp and biofuels.

**Results:**

A comprehensive in silico analysis of family GH11 sequences from the CAZY database allowed their phylogenetic classification in a radial cladogram in which sequences of known or presumptive thermophilic and alkalophilic xylanases appeared in three clusters. Eight sequences from these clusters were selected for experimental analysis. The coding DNA was synthesized, cloned and the enzymes were produced in *E. coli*. Some of these showed high xylanolytic activity at pH values > 8.0 and temperature > 80 °C. The best enzymes corresponding to sequences from *Dictyoglomus thermophilum* (Xyn5) and *Thermobifida fusca* (Xyn8). The addition of a carbohydrate-binding module (CBM9) to Xyn5 increased 4 times its activity at 90 °C and pH > 9.0. The combination of Xyn5 and Xyn8 was proved to be efficient for the saccharification of alkali pretreated rice straw, yielding xylose and xylooligosaccharides.

**Conclusions:**

This study provides a fruitful approach for the selection of enzymes with suitable properties from the information contained in extensive databases. We have characterized two xylanases able to hydrolyze xylan with high efficiency at pH > 8.0 and temperature > 80 °C.

## Background

Xylan, the most abundant type of hemicellulose, is a polysaccharide composed by a linear backbone of β-1, 4-linked xylose units. Together with cellulose and lignin, xylan is one of the main constituents of plant cell walls. Xylan degradation into simple sugars, a preliminary step for its conversion into different bioproducts, is carried out by the concerted action of different xylanolytic enzymes [1, 2]. Xylanases produced by many microbial species are an important type of industrial enzymes with multiple applications. They are used as additives to enhance the quality of baked goods [3] and animal feeds [4], as well as to bleach kraft pulp [5, 6].

Enzymatic biotransformation of xylan is limited by different factors such as the nature of the substrate, physicochemical conditions (pH, temperature), presence of inhibitors and cost of enzyme product. Xylan structure is highly variable between different plant species. The poly-xylose backbone often presents a complex branching pattern that may represent a steric limitation for the xylanases to reach their target [7, 8]. Xylanases and xylosidases play the most important role in depolymerization of the xylan backbone, while other enzymes act on the cleavage of the side chains [9].

Based on the structural criteria, most xylanases are classified into two glycoside hydrolase (GH) families, 10 and 11; whereas, enzymes with xylanolytic activity are also present in GH families 5, 8, 16, 18, 26, 30, 43, 48, 51, 52, 62, 98 and 141 [10]. Family GH11 comprises enzymes characterized by a small (< 30 kDa) catalytic domain with β-jelly roll structure, with various activities, including xylanase (EC 3.2.1.8) and endo-1,3-β-xylanase (EC 3.2.1.32). Xylanases of this family are considered “true xylanases” acting on xylan with high specificity. Their properties: high substrate selectivity, catalytic efficiency, small size and wide range of optimum pH and temperature values, make them useful for different industrial applications [11].

The pulp and paper industry is an important application niche for xylanases [5, 6]. Current growing concern to decrease pollution drives the substitution of chemical technology by environmentally friendlier enzymatic procedures. In this context, biobleaching and biopulping processes have been explored over the past years. It has been shown that the use of xylanases may be an economically profitable alternative to conventional use of high amount of chemicals, which cause hazardous effluent disposal problems [12–15].

Economically viable implementation of an enzyme-based technology in the pulp and paper industry requires the production of enzymes active at the extreme conditions of alkaline pH and temperature used in wood processing. In the past years, sequencing of the genomes of an increasing number of microbial species and metagenomic analysis have produced a plethora of data from which valuable information can be obtained. Therefore, to find enzymes with suitable properties, we have undertaken a comprehensive bioinformatic survey of family GH11 amino acid sequences retrieved from publicly available databases. Our final goal was to set up a predictive tool to identify putative thermophilic, alkaliphilic xylanases. Since glycoside hydrolases frequently have a modular composition, including a variety of non-catalytic domains fused to the catalytic module, the domain architecture (DA) of GH11 sequences was analyzed in the first place, to identify non-catalytic modules appended to the GH11 catalytic core, which may add a significant functional role to xylanase performance. Second, a phylogenetic analysis, using the Glyco_hydro_11 (PF00457) (catalytic) domain extracted from the GH11 sequences, was carried out. This cladogram resulting from this analysis is expected to provide information about the effect of the accompanying non-catalytic modules in the evolution of GH11 proteins and reveal clusters of enzymes with similar properties (thermal/alkaline stability in our case). In the last term, this analysis should provide a fair prediction of the functional properties of a given putative enzyme sequence, even without knowing its source. To test the validity of our approach, we carried out the functional characterization of selected sequences from ‘alkaliphilic, thermophilic’ regions defined by the in silico analysis, under extreme conditions of pH and temperature. We also present the results of the prospective application of the more active enzymes under the defined conditions, for the hydrolysis of rice straw xylan.

## Results

### Phylogenetic analysis of the GH11 family

Phylogenetic analysis of the GH11 family was carried out using the Glyco_hydro_11 (GH11) Pfam domain that contains the catalytic residues and is the characteristic motif of the enzymes of this family. From the total number of GH11 sequences listed in the CAZy database, 1306 sequences were analyzed, after discarding those showing coverage lower than 80% of the consensus sequence of the GH11 domain. Selected sequences could be classified into 62 different domain architectures (DAs) belonging to eukarya (tagged with an E), prokarya (not tagged) and archaea (tagged with an A) (Table 1). In all 1306 sequences (with two exceptions), the GH11 domain was present at the N-terminus. In both prokaryotes and eukaryotes, a majority of the sequences (ca. 70 and 80%, respectively) were composed by the simplest type of DA, consisting of a GH11 domain alone. Around 15% of the sequences, from both types of organisms, displayed a carbohydrate-binding domain (CBM) following the GH11 domain. CBM4-9, CBM6 and CBM60 were the most frequent among prokaryotes, whereas CBM1 and CBM10 were found in eukaryotes. Other types (CBM2, CBM5–12–2 and CBM9) could also be found. A significant fraction of prokaryotic sequences (ca. 12%) and some of the eukaryotic (ca. 3%) contained C-terminal extensions with unidentified Pfam motifs. These C-terminal tails were labeled according to their length as Ct1 (50–150 aa), Ct2 (150–200 aa) or Ct3 (200–300 aa). The different protein domains detected in GH11 sequences are listed in Additional file 1: Table S1.

**Table 1:**
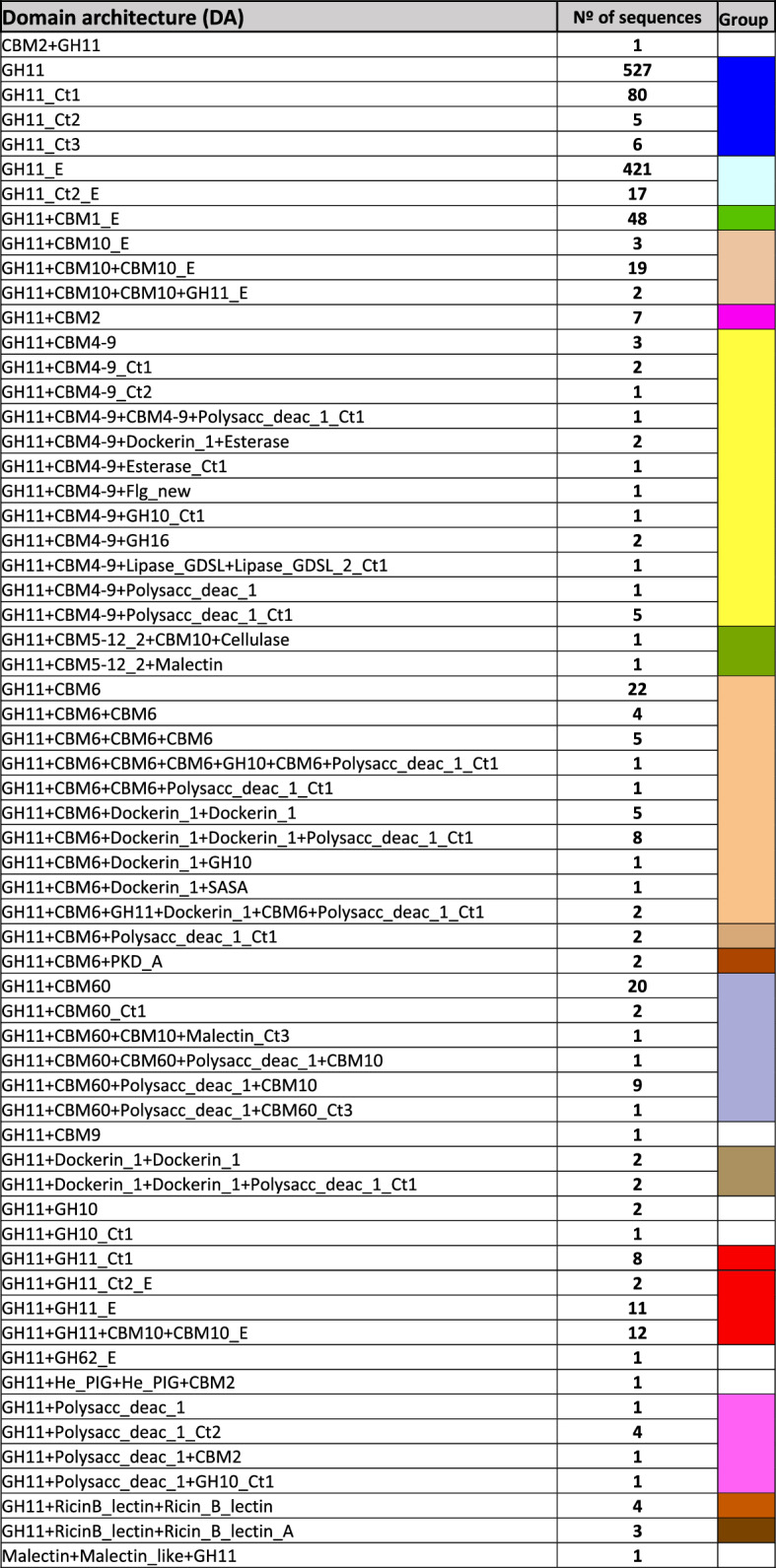
Classification of Domain Architectures (DA) found in GH11 protein sequences

**Fig. 1:**
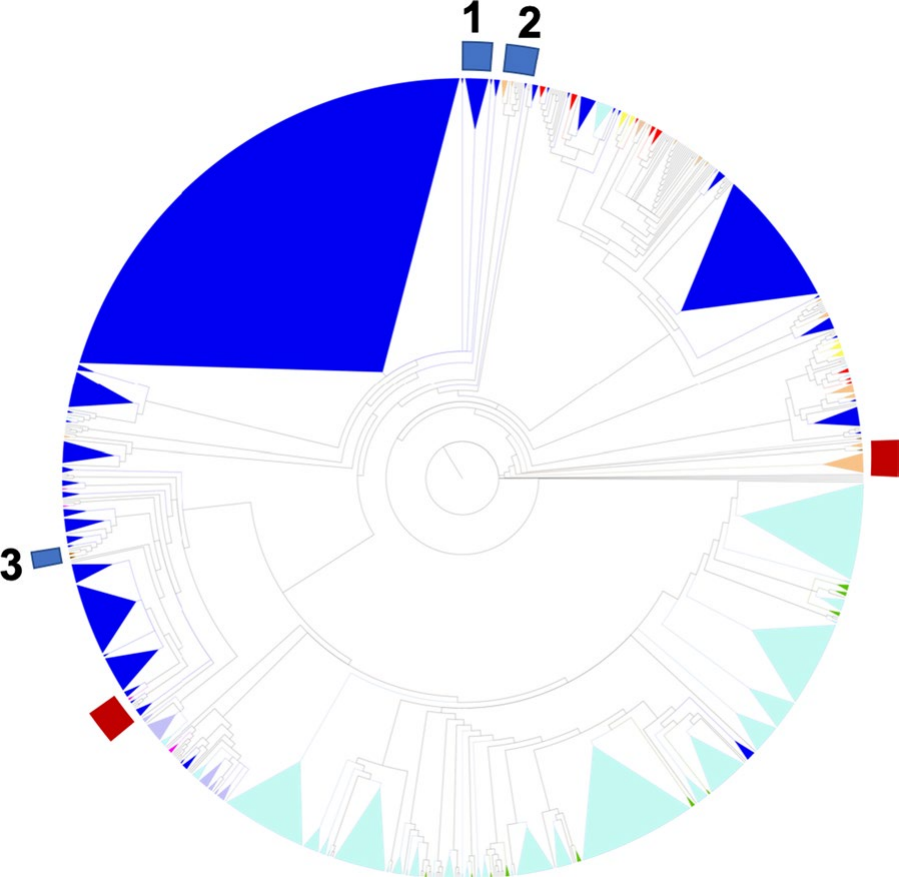
Cladogram of Family GH11 (GH11 domain) sequences. Clusters depicted in blue, labeled 1, 2 and 3 include sequences that may correspond to xylanases active in alkaline conditions and high temperature. Clusters depicted in red contain sequences corresponding to acidophilic, thermoresistant xylanases. Color code of the sequence clusters corresponds to that used in Table 1 for the different domain architectures

The results of phylogenetic analysis are shown schematically as a radial cladogram (Fig. 1). The complete list of analyzed sequences and a full-size version of the cladogram are shown in Additional files 2 and 3. Different DA types are represented by different colors as indicated in Table 1. As it was expected, eukaryotic sequences cluster together, springing from neighbor branches, at a late stage in the evolutionary process, although they do not arise from a single node, indicating that were originated by different evolutionary events. The simplest DA, composed by one GH11 domain appears not only at early stages of evolution but also at later stages, associated with more complex DAs. In a few cases, addition of C-terminal domains did not result in a distinct evolution of the catalytic domain, since both topologies were grouped within the same node (Fig. 1). However, most sequences with additional domains are grouped in nodes separated from those with the simplest topology.

**Fig. 2:**
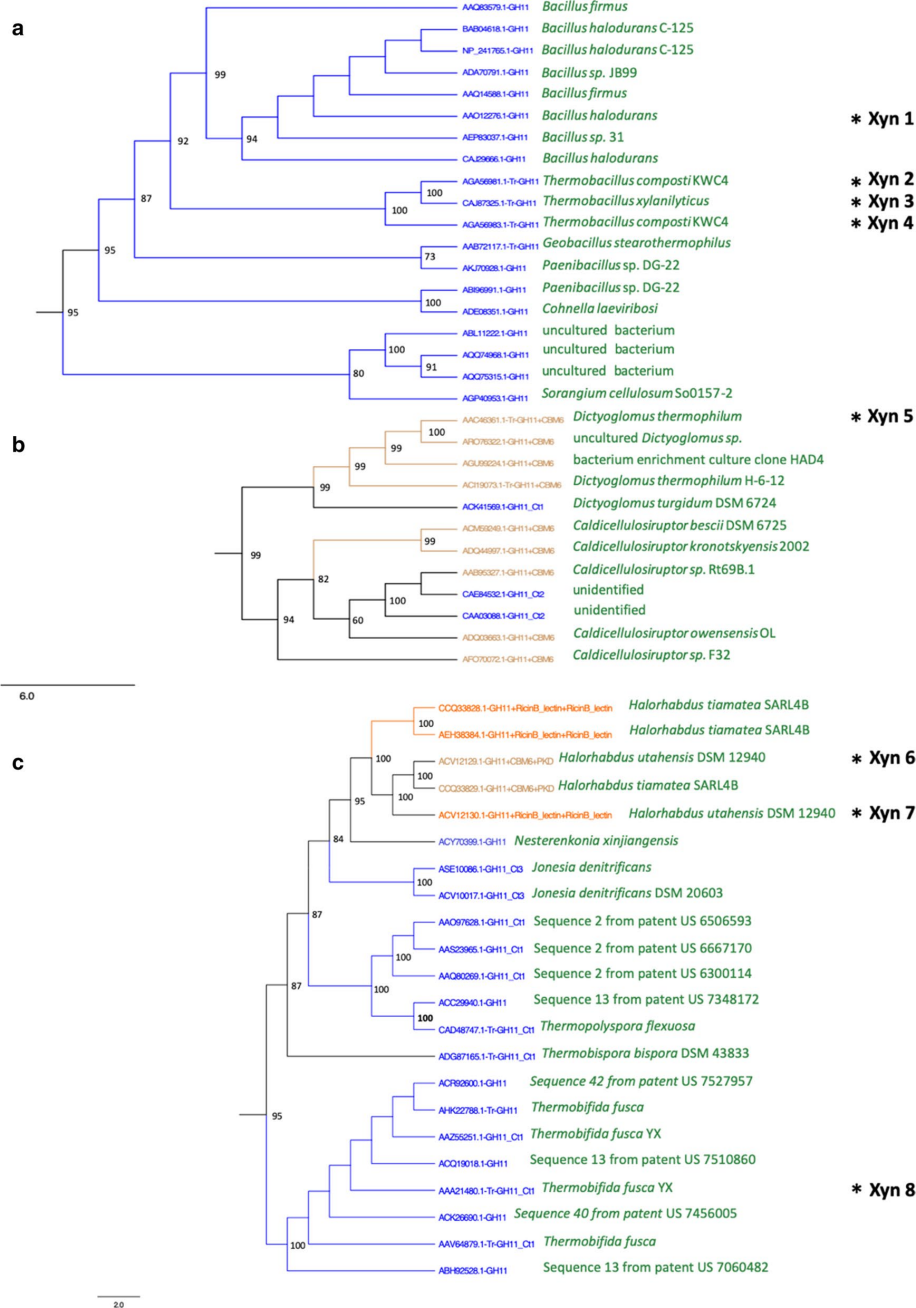
Phylogenetic subtree corresponding to clusters 1 (**a**), 2 (**b**) and 3 (**c**) of the GH11 circular cladogram. Branch numbers indicate bootstrap values, absence of bootstrap values indicate < 50 and are not considered as significant. Sequences selected for experimental analysis are marked by an asterisk, indicating their alternative (short) designation. Color code of the sequences is the same used in Table 1 for the different domain architectures

Putative thermophilic xylanases (labeled “Tr”; Additional file3: Figure S1) appeared grouped in five clusters in the cladogram (Fig. 1). It was found that clusters 1, 2 and 3 often corresponded to alkalophilic organisms; whereas, the others included acidophilic organisms, outside the scope of this study. Cluster 1 (Fig. 2a) contained sequences from thermophilic microorganisms, including *Bacillus, Thermobacillus,* and *Geobacillus*, all of them showing the simplest domain architecture GH11. Cluster 2 (Fig. 2b) included sequences from *Dictyoglomus* and *Caldicellulosiruptor* and corresponded to DAs with a CBM6 module attached to the catalytic domain. Unidentified modules labeled as Ct1 and Ct2 within this cluster were further analyzed with the Interpro tool [16] and revealed that they corresponded to a CBM6 domain (IPR005084) in all cases. Cluster 3 (Fig. 2c) contained not only thermophilic sequences from *Thermopolyspora, Thermobifida,* and *Halorhabdus*, but also mesophilic such as those from *Nesterenkonia* or *Jonesia*. Most putative xylanases in this cluster presented the simplest DA (GH11) or contained unidentified C-terminal tails (Ct1 or Ct3). However, DAs with simplest topology often were patented sequences that could have been manipulated, not representing natural structures. Ct1 and Ct3 extensions contained one or two CBM2 (IPR001919), respectively, according to Interpro analysis. Sequences from *Halorhabdus* had DAs which are less frequent in the GH11 family, either with two Ricin-type lectin-like domains or with a CBM6 fused to a PKD domain. The Ricin_type lectin-like domain is recognized in the CAZy database [10] as a CBM13, which has shown xylan-binding activity in xylanases [17]; whereas, the PKD domain belongs to the superfamily of modules with an immunoglobulin-like fold (IPR013783). Seven sequences from these three clusters, representing different DAs, were selected for experimental analysis (Xyn1-8). These sequences corresponded to *Bacillus halodurans*, *Thermobacillus composti*, *Dictyoglomus thermophilum*, *Halorhabdus utahensis* and *Thermobifida fusca* (Table 2). Although three of these enzymes, Xyn3 [18], Xyn5 [19] and Xyn8 [20] have been previously analyzed, they were included in our study, to be compared with the others under the same assay conditions.

**Table 2:**
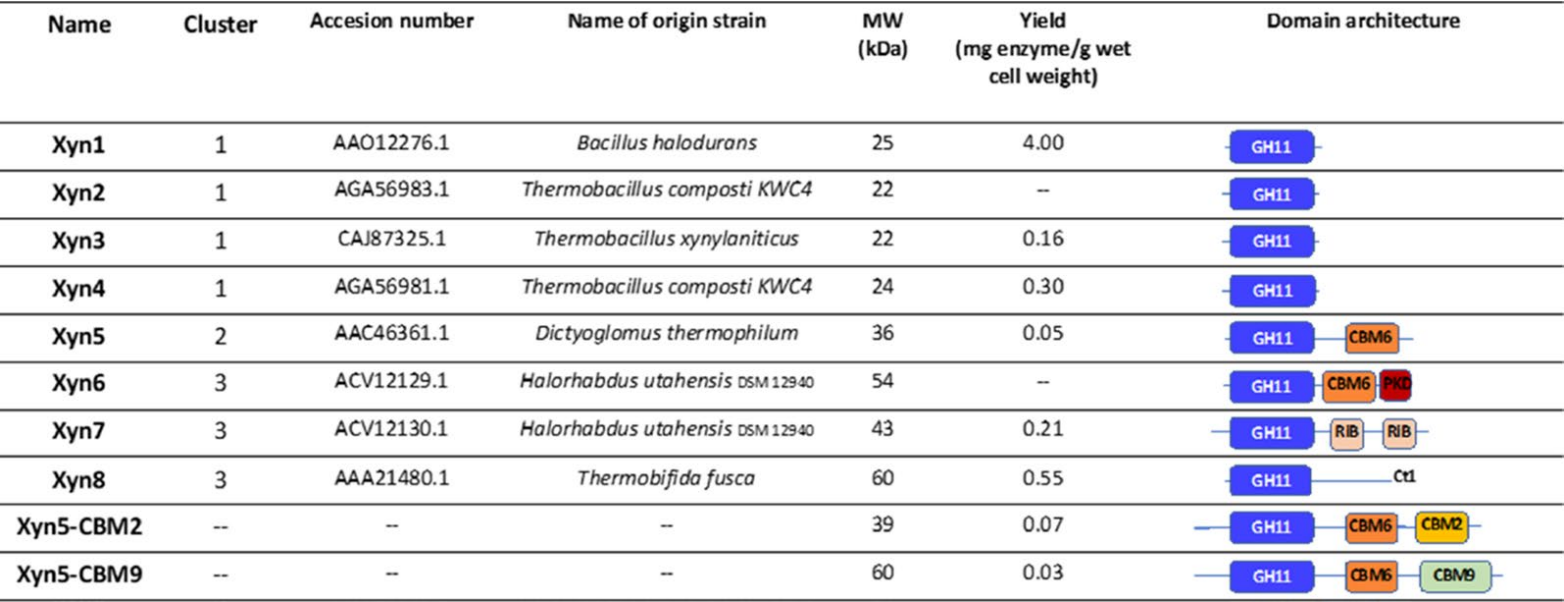
Putative thermophilic, alkaliphilic protein sequences selected from the *in silico *analysis for functional characterization

### Production and purification of putative xylanases

**Fig. 3:**
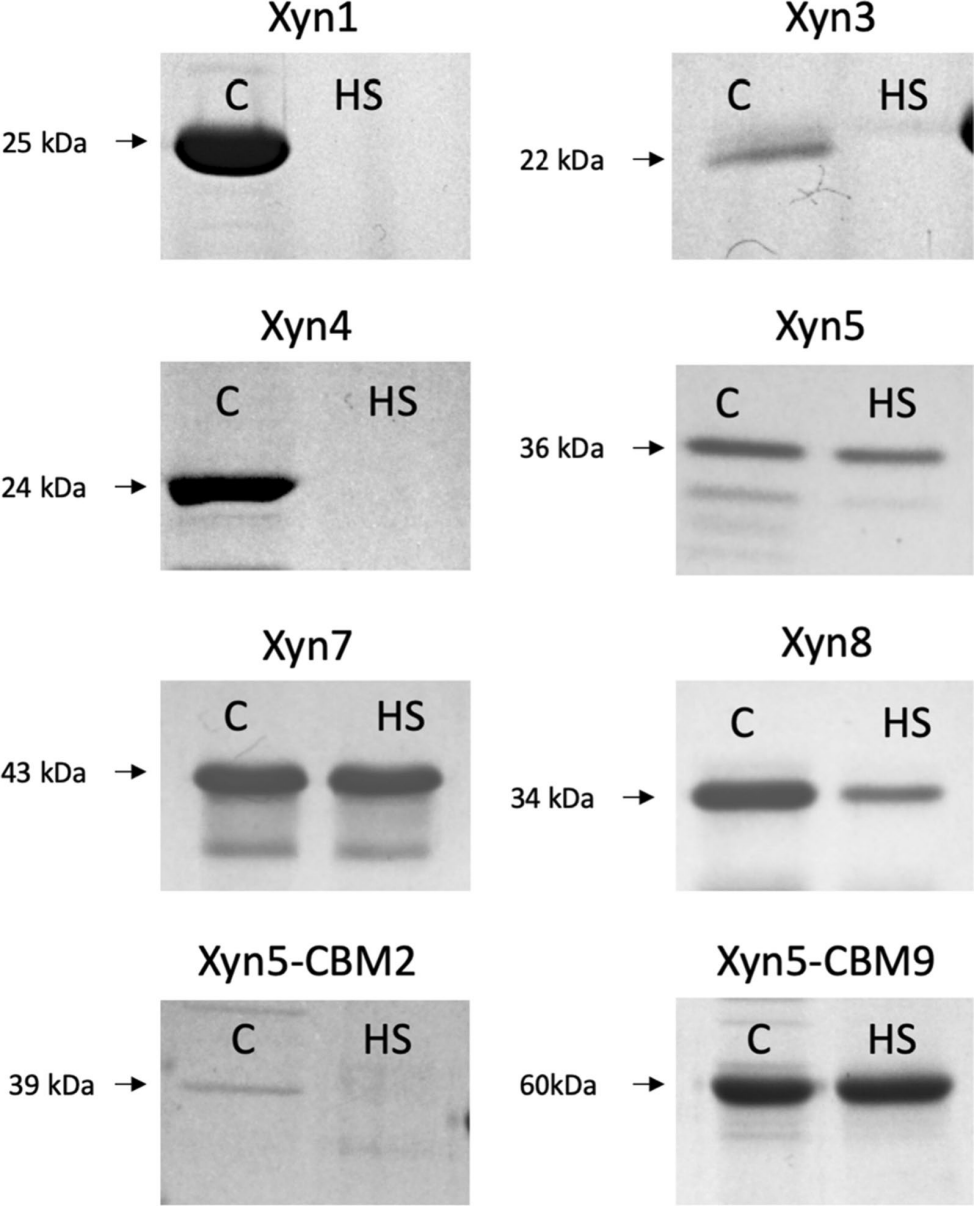
SDS-PAGE of xylanases purified by nickel-affinity chromatography, before (**c**) and after (HS) thermal treatment at 85 °C, 5 min

Synthetic, codon-optimized gene sequences were expressed in *E. coli* to produce the selected putative xylanases. Additionally, to analyze the possible effect of carbohydrate-binding modules, chimeric proteins were constructed by fusion of Xyn5 to CBM2 from *Pyrococcus furiosus* [21] (Xyn5–CBM2) and CBM9 from *Thermotoga maritima* [22] (Xyn5–CBM9) (Table 2). Synthesis of the coding sequences of xylanases was accomplished. Xyn2 was discarded at this point because we noticed that it was identical to Xyn3, except for the presence of a signal peptide in Xyn2. Xyn6 could not be synthesized in one single fragment and has to be obtained by assembling two synthesized fragments (Xyn6-F1 and Xyn6-F2). Sequencing revealed the existence of a gap of 36 base pairs in fragment Xyn6-F1. Repeated synthesis and cloning attempts led to the same result, likely due to the existence of a guanine-rich stretch at the gap region. Therefore, Xyn6 was finally excluded from further analysis. *E. coli* transformant cultures carrying plasmids with genes encoding the different xylanases were induced with IPTG. This led to the production of His-tagged versions of the enzymes that were purified from bacterial crude cell extracts by nickel-affinity chromatography. Xylanases were obtained with different yields after purification (Table 2). Thermal stability was analyzed by heating purified proteins at 85 °C for 5 min. The soluble fraction recovered after this treatment was analyzed by SDS-PAGE, in parallel to the untreated samples (Fig. 3). In all cases, electrophoretical mobility was in concordance with the expected molecular mass of the xylanases, predicted by ProtParam [23]. Highest thermal stability was shown for Xyn5 and Xyn7: most protein remained soluble and enzymatically active after heat treatment. Protein from Xyn1, Xyn3, Xyn4 and Xyn8 precipitated after heating. Hybrid enzyme Xyn5–CBM9 was thermostable and Xyn5–CBM2 was unstable.

### Enzymatic activity of putative xylanases

**Fig. 4:**
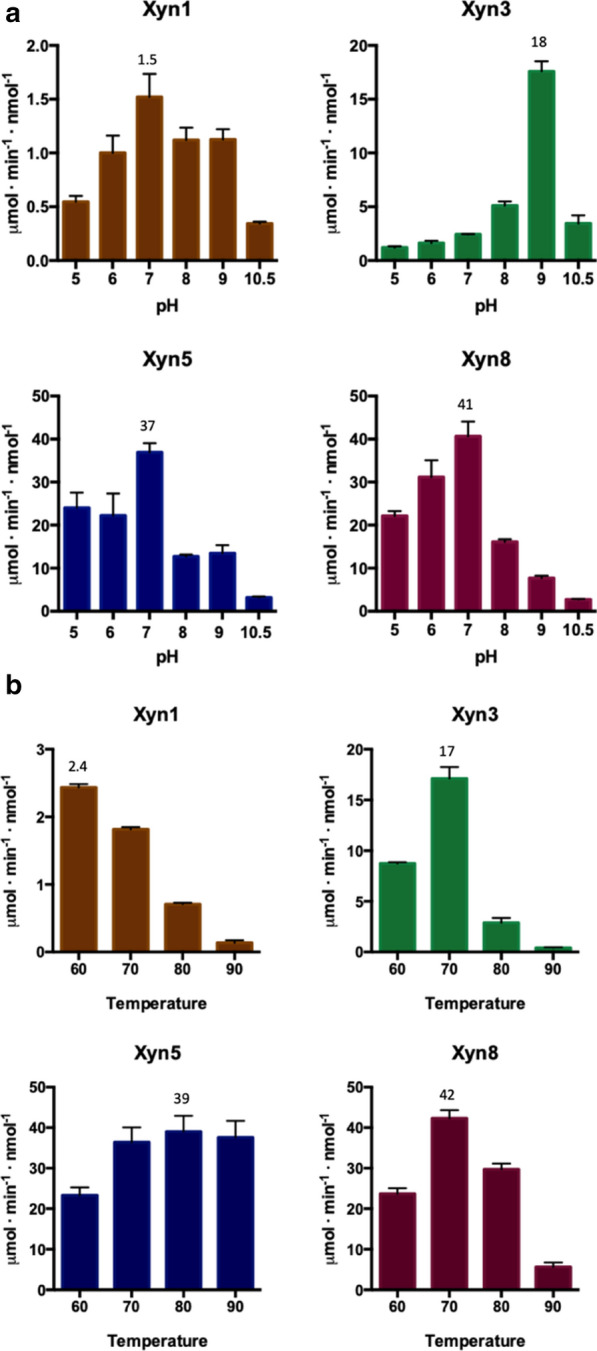
Activity assay of xylanases Xyn1, Xyn3, Xyn5 and Xyn8 with 1% oat-spelt xylan as the substrate, carried out by measuring reducing sugars with the DNS method. Assays at different pH values (**a**) were conducted at 65 °C. Assays at different temperatures (**b**) were conducted at pH 9.0. Error bars indicate standard deviation of triplicates

**Fig. 5:**
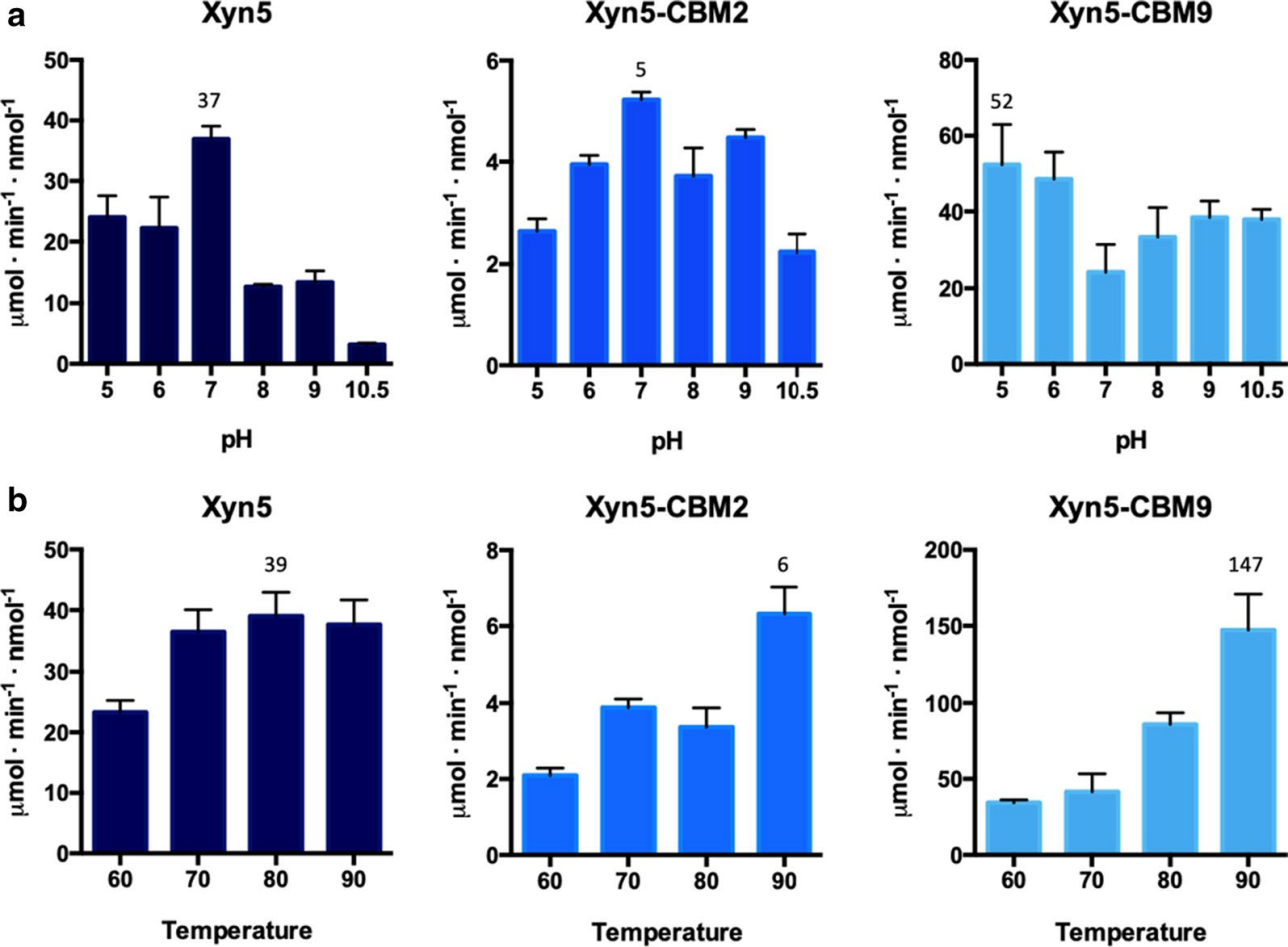
Activity assay of Xyn5 and hybrids Xyn5–CBM2 and Xyn5–CBM9 with 1% oat-spelt xylan as the substrate, carried out by measuring reducing sugars with the DNS method. Assays at different pH values (**a**) were conducted at 65 °C. Assays at different temperatures (**b**) were conducted at pH 9.0. Error bars indicate standard deviation of triplicates

Preliminary semiquantitative activity determination showed that Xyn4 and Xyn7 were clearly less active than the other enzymes and, therefore, were discarded. Xylan hydrolysis by Xyn1, Xyn3, Xyn5 and Xyn8 was measured at different pH and temperature. Assays at different values of pH were carried out at 65 °C (Fig. 4a). Only Xyn3 showed the profile of an alkalophilic enzyme, with optimal activity at pH 9.0. Xyn1, Xyn5 and Xyn8 showed optimal activity at pH 7.0. Xyn1 was active at a wider range of pH, keeping ca. 70% of its maximal activity at pH 9.0; while, Xyn5 and Xyn8 retained only 40% and 20% of optimal activity at this pH. The enzymatic activity of the xylanases was assayed at different temperatures (in the range 60–90 °C), in buffered solution at pH 9.0. Xyn5 was the most thermophilic enzyme, retaining maximal activity between 70 and 90 °C (Fig. 4b). Optimal temperatures of Xyn1 (60 °C), Xyn3 (70 °C) and Xyn4 (70 °C), were significantly lower. Taking together the pH and temperature profiles, Xyn5 yielded the best results, showing high activity at pH 9.0 and 90 °C. On the basis of these results, hybrid enzymes Xyn5–CBM2 and Xyn5–CBM9 were analyzed. Addition of the CBM2 domain caused a decrease of Xyn5 xylanase activity of about seven times. The hybrid enzyme had the same optimal values of pH (7.0) and temperature (90 °C) as the parental Xyn5 (Fig. 5). Fusion of Xyn5 to CBM9 had two remarkable effects. Optimal pH, measured at 65 °C, was displaced from 7.0 to ≤ 5.0, although relatively high values of activity were maintained even at very alkaline conditions (pH 10.5) (Fig. 5a). Even more remarkably, the addition of CBM9 increased enzyme activity at high temperature, by a factor of about four times at 90 °C (Fig. 5b).

### Effect of xylanases on the saccharification of rice straw

**Fig. 6:**
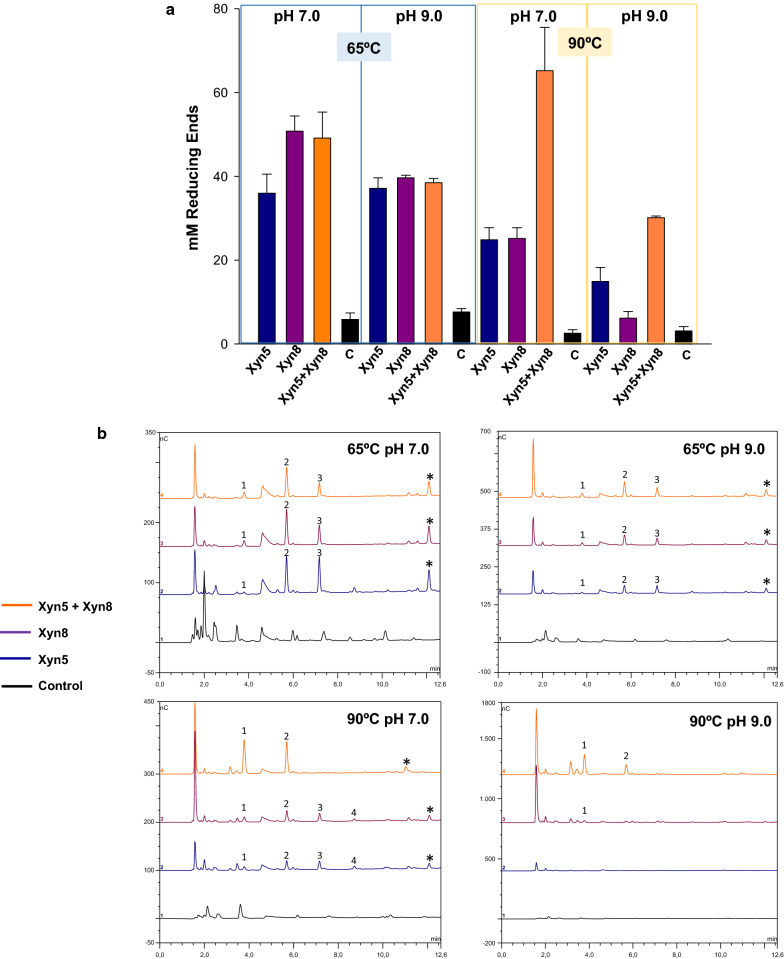
Analysis of reducing sugars after application of xylanases to alkali pre-treated rice straw. **a** Reducing sugars were measured by the DNS method, after 24-h digestion at the indicated conditions of pH and temperature. C (Control) corresponds to treated rice straw not subjected to enzyme treatment. Error bars represent standard deviation among three replicates. **b** Chromatographic profile of products obtained after application of xylanases to alkali-treated rice straw, as before. Peaks 1, 2, 3 and 4 correspond to xylose, xylobiose, xylotriose and xylotetraose, respectively. Other relatively abundant, unidentified products are marked with an asterisk

**Fig. 7:**
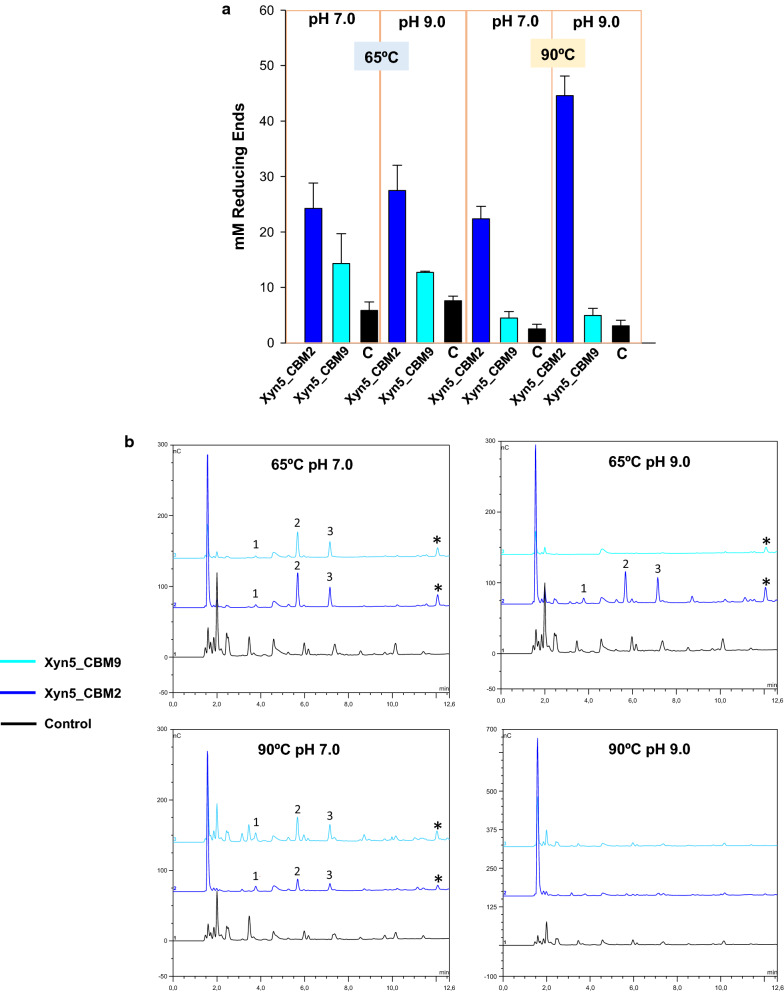
Analysis of reducing sugars after application of hybrid xylanases Xyn5–CBM2 and Xyn5–CBM9 to alkali pre-treated rice straw. **a** Reducing sugars were measured by the DNS method, after 24 h digestion at the indicated conditions of pH and temperature. C (Control) correspond to treated rice straw not subjected to enzyme treatment. Error bars represent standard deviation among three replicates. **b** Chromatographic profile of products obtained after application of xylanases to alkali-treated rice straw, as before. Peaks 1, 2 and 3 correspond to xylose, xylobiose, and xylotriose, respectively. Other relatively abundant, unidentified products are marked with an asterisk

Xylanases that showed better performance in extreme conditions were selected to analyze their efficiency for the digestion of rice straw. Soluble sugars released by the action of Xyn5, Xyn8 and a mixture of Xyn5 and Xyn8 on rice straw, pretreated with alkali at high temperature, were measured. The highest production of reducing sugars (ca. 70 mM) was obtained at pH 7.0 and 90 °C, using a combination of the two xylanases, showing a synergistic effect of the two enzymes (Fig. 6a). Considering rice straw was at a concentration of 10% (w/v) and that the hemicellulose content in this substrate ranges between 18 and 25% [24], the maximal theoretical amount of reducing sugars (up to monosaccharides) would be around 151 mM. Thus, the effect of the combined action of the two xylanases at pH 7.0 and 90 °C represents a significant transformation of rice straw, with ca 50% of the theoretical maximum achieved. Chromatographic analysis (Fig. 6b) showed production of xylose and xylooligosaccharides (2–4 units) together with less abundant unidentified products, as it would be expected from the digestion of a complex substrate. This result agreed with what was expected from the endo-acting mechanism of GH11 xylanases. In accordance with the measurement of reducing sugars, the highest level of saccharification (maximum production of xylose) was achieved with simultaneous treatment with the two xylanases, at pH 7.0 and 90 °C. Production of soluble sugars and chromatographic analysis was also carried out for hybrid enzymes Xyn5–CBM2 and Xyn5–CBM9. Xyn5–CBM2 became more effective that Xyn5–CBM9 in all conditions, particularly at 90 °C and pH 9.0 (Fig. 7a). The pattern of soluble sugars produced by the digestion of rice straw with the hybrid enzymes was similar to the incomplete saccharification obtained with Xyn5 and Xyn8, with the detection of xylose and relatively high amounts of xylobiose and xylotriose, indicating incomplete digestion.

## Discussion

In silico screening of sequence databases provides a powerful methodology for the search of enzymes with specific properties [25, 26]. In this work, we have undertaken a bioinformatics analysis of family GH11 of glycoside hydrolases, that includes one of the major of ensemble of xylanases [27], aiming to the identification of extremophilic enzymes active at strongly alkaline pH and high temperature. This type of analysis is less laborious and allows wider-range hunts than experimental approaches based on cloning and screening of large number of genes [28]. Diverse bioinformatic tools have been described for enzyme identification and phylogenetic analysis of different types of glycoside hydrolases from genome or protein databases [23, 29, 30].

GH11 sequences could be classified in two large groups, considering their domain architecture (DA). The first, simplest type contained just the catalytic GH11 domain; whereas in the other type, the GH11 domain was linked to another (occasionally more than one) domain, most frequently a CBM. Non-catalytic domains condition enzyme activity since they determine the affinity for a certain substrate. Moreover, they are part of the protein structure and, therefore, affect enzyme stability and activity at different conditions of pH and temperature. CBM1 is almost found exclusively in fungi and binds to cellulose and xylan [31]. Despite its presence in xylanases, CBM10 has been characterized as a cellulose-binding module [32]. CBM4 and CBM6 are related modules, with the same B-type topology and affinity for xylan [33, 34]. CBM9 [35] and CBM60 [36] have xylan-binding activity and are characteristic of xylanases. Available information about CBM5 is scarce. It is mostly found in bacteria, has chitin-binding activity and is present in non-hydrolytic enzymes, e.g., monooxygenases [37]. Non-catalytic domains, other than CBMs appear associated with GH11. The dockerin domain is found in proteins associated with cellulosomes [38]. Malectin [39] and Ricin B lectin domain [40] have carbohydrate recognition function. GH11 is also found associated with other catalytic domains active against different constituents of the plant cell wall, such as the polysaccharide deacetylase domain [41], the esterase domain [42] and the lipase GDSL domain [43].

Phylogenetic classification of family GH11 sequences in a cladogram revealed the existence of three clusters that presumptively corresponded to the pre-established requirements of xylanases active in alkaline conditions and high temperature. The sequences selected using this approach and in particular three of them (Xyn3, 5 and 8), once synthesized and characterized, fulfilled the expectations. All four xylanases showed high activity at pH 9.0. Xyn3 from *Thermobacillus xylanilyticus* had been studied previously [18] but the enzyme was assayed at pH 5.8 and not described as alkalophilic. Overall, two enzymes, Xyn5 from *Dictyoglomus thermophilum* [19] and Xyn8 from *Thermobifida fusca,* showed exceptionally good results [20].

Analysis of GH11 family sequences shows that about 15% contain a CBM. The existence of a given CBM determines the affinity of the enzyme for a particular polysaccharide and consequently the enzyme properties from a biotechnological point of view. Xyn5, the enzyme that showed better properties in this analysis, carries a CBM6. To further the study of CBMs function, we constructed hybrid enzymes Xyn5–CBM2 and Xyn5–CBM9, using thermostable modules CBM2 from *Pyrococcus furiosus* chitinase [21] and CBM9 from *Thermotoga maritima* xylanase [22]. We decided not to eliminate CBM6 since its deletion would likely have adverse effects on protein stability. Assays of enzyme activity with oat-spelt xylan as the substrate, under extreme conditions of pH and temperature, showed that CBM2 decreased enzyme performance whereas CBM9 improved considerably the activity. It is difficult to explain the structural basis of the observed effect since it depends not only of the type of CBM but also of other factors, such as the interrelationship between the catalytic module and the CBM, and the composition of substrate.

Xylanases have a great potential for the bioconversion of plant waste material into bioethanol and other products [44]. The criteria used in this work for xylanase selection were enzyme compatibility with conditions of alkaline pH and high temperature widely used in the pretreatment of plant material. Therefore, we have assayed the performance of selected xylanases in the saccharification of xylan under similar conditions, using rice straw as substrate. Currently, rice straw is an agricultural waste that generates considerable environmental problems for its elimination. However, it can be a suitable raw material for bioethanol production [44, 45]. Treatment with enzymes Xyn5, Xyn8 and hybrids Xyn5–CBM2 and Xyn5–CBM9 yielded xylose and oligoxylosides from the rice straw. With rice straw as the substrate, Xyn5–CBM2 performs better than Xyn5–CBM9; whereas, the opposite is true when purified xylan was used as substrate. This can be explained by differences in both the structure of the CBMs and the chemical composition of the substrates. CBM2 is a small domain with a planar-binding surface that binds cellulose [21]; these properties would facilitate its interaction with xylan-rich rice straw cell wall material. Xyn5–CBM9, carrying a larger, pocket shaped CBM [22] would interact better with purified xylan chains.

Best saccharification results were obtained with a combination of Xyn5 and Xyn8. The synergistic effect of these two enzymes may be due to different substrate specificities in their catalytic and/or non-catalytic domains. In the experimental conditions explored in this study, hydrolysis of rice straw reached ca. 50% of the maximum theoretical conversion of xylan to xylose.

## Conclusions

We have devised a bioinformatic approach to identify putative xylanases with desired properties (i.e., good performance under conditions of alkaline pH and high temperature) among ca. 1800 family GH11 entries available in the CAZY database. The bioinformatics analysis rendered a phylogenetic cladogram in which putative sequences of thermophilic and alkalophilic xylanases, with different domain architecture, appeared grouped in three clusters. Eight sequences from these clusters were selected for an experimental analysis that allowed the identification of two xylanases, one from *Dictyoglomus thermophilum* (Xyn5) and another from *Thermobifida fusca* (Xyn8), which showed the best properties. These enzymes showed efficient xylan degrading activity at pH > 8.0 and temperature > 80 °C. The effect of carbohydrate-binding domains (CBM) on enzyme function was investigated using two hybrid proteins: Xyn5–CBM2 and Xyn5–CBM9, which were constructed by the addition of thermostable CBM modules. Whereas CBM2 had a negative effect, CBM9 improved enzyme activity regarding both high temperature and alkaline pH. This result is relevant from a practical point of view since CBM9 addition increased enzyme activity by 2–3 times, at 90 °C and pH 9.0–10.5. Digestion of alkali pretreated rice straw with enzymes Xyn5, Xyn8 and hybrids Xyn5–CBM2 and Xyn5–CBM9 yielded substantial amounts of xylose and oligoxylosides. Best results, with increased production of xylose, were achieved by using Xyn5 and Xyn8 in combination, revealing a synergistic effect of the two enzymes.

## Methods

### Analysis of protein domain architectures

GenBank accession numbers of protein sequences of family GH11 were obtained from the CAZy database [10] and their amino acid sequences retrieved from the NCBI database, using the Batch Entrez Tool (https://www.ncbi.nlm.nih.gov/sites/batchentrez). For each sequence, protein domain composition and coordinates were determined using Pfam [46]. Domain architecture (DA) is defined by the linear composition of domains of a given sequence in N-terminal to C-terminal order. Classification of GH11 sequences into specific DAs was carried out following the same methodology previously described for the GH2 family [25]. Among the GH11 sequences listed in the CAZy database, those containing a catalytic domain matching at least 80% the Pfam consensus signature (PF00457) were retrieved. These sequences were processed to extract the catalytic domain sequence Glyco_hydro_11 (PF00457) that was used to perform the phylogenetic analysis.

### Phylogenetic analysis

Sequence alignment of GH11 catalytic domains was performed with CLC sequence viewer (Qiagen), using Clustal Omega MSA algorithm [47]. Trees were built using W-IQ-TREE Maximum Likehood algorithm with JTT matrix  [48] and a bootstrap of 1000 replicates. Results were analyzed on Dendroscope Software  [49] and represented using FigTree software (http://tree.bio.ed.ac.uk/software/figtree/).

Sequences present in C-terminal position in some domain architectures, not identified by Pfam, were considered in this analysis and labeled as a function of their size as Ct1 (50–150 aa), Ct2 (150–200 aa), Ct3 (200–300 aa), Ct4 (300–400 aa) and Ct5 (400–500 aa). Taking into consideration available information about the taxonomic genera to which the sequences belong, DA were tagged as putative thermostable (Tr), putative alkalophilic (Ak) and putative acidophilic (Ac).

### In silico sequence edition

Amino acid and DNA sequences chosen for experimental analysis were edited before cloning. Signal peptide was detected using the Phobius Tool [50] and removed. The coding sequences were optimized for *E. coli* expression by using the Integrated DNA Technologies (IDT) Codon Optimization Tool (www.idtdna.com). Native restriction sites were eliminated and *Sac*I and *Sal*I restriction sites were added in 5′ and 3′, respectively, to facilitate the cloning in vector pQE-80L (Quiagen).

### Molecular biology techniques

Synthetic, codon-optimized, genes of sequences encoding seven selected putative xylanases (Xyn1-8) were purchased from IDT (Additional file 4: Table S3). The DNA fragments (except Xyn6) were digested with endonucleases *Sac*I and *Sal*I and cloned into pQE80L plasmid cut with the same enzymes. Xyn6 could not be synthesized as a single piece and was obtained in two fragments: Xyn6-F1, which was cut with *Sac*I and *Kpn*I, and Xyn6-F2 cut with *Kpn*I and *Sal*I and then cloned in pQE80L. Fast Digest enzymes and T4 ligase were purchased from ThermoScientific. The resulting plasmids were transformed in to *E. coli* XL1Blue, and selected for protein purification in *E. coli* Rosseta (Stratagene). Hybrid enzymes Xyn5–CBM2 and Xyn5–CBM9 were constructed from plasmids TmLac–CBM2 PQE80L and TmLac–CBM9  [51], respectively, replacing the TmLac gene by the Xyn5 gene.

### Selection of *E. coli* clones expressing xylanase genes

Screening of clones with xylanase activity was carried out in 96-well cell culture plates. Transformant *E. coli* colonies were grown in 200 μL of LB with 100 mg/L ampicillin at 37 °C, with shaking, 180 rpm, overnight. A volume of 10 μL from each well was transferred to a new plate with fresh LB supplemented with ampicillin, which was further incubated until the cultures reached a cell concentration of about 0.6 OD_600_. The cultures were induced with IPTG 1 mM at 16 °C overnight. The cells were collected by centrifugation, resuspended in 150 μL of lysis buffer (phosphate buffer 50 mM pH 7.0 with 2.5 mg/mL lysozyme) and incubated for 1 h at 37 °C. A volume of 20 μL of each cell crude extract was added to 180 μL of oat-spelt xylan 1% (Sigma) in phosphate buffer 50 mM pH 6.5 and incubated at 65 °C for 30 min. Xylanase activity was determined by measuring reducing sugars resulting from the enzyme action. For this purpose, 100 μL of DNS (dinitrosalicylic acid) reagent (Sigma) was added to each reaction well and the plate was incubated for 30 min at 85 °C. Positive clones were identified by a dark orange color. The identity of the coding sequence responsible for the activity of the xylanolytic clones was checked by DNA sequencing.

### Protein purification

Cell crude extracts were prepared from *E. coli* cultures grown at 37 °C up to a cell density (OD_600_) of 0.6 and induced with 1 mM IPTG, either at 16 °C overnight or 37 °C for 5 h. The cells were disrupted by sonication in buffer A (20 mM phosphate buffer, pH 7.4, 10 mM imidazole, 500 mM NaCl). Protein extracts were recovered by centrifugation at 12,000×g during 25 min and subjected to nickel-affinity chromatography using 1 mL HisTrap FF crude column (GE Healthcare) mounted in AKTA-Purifier (GE), using buffer B (20 mM phosphate buffer, pH 7.4, 500 mM imidazole, 500 mM NaCl) for elution. Eluted fractions showing xylanase activity were dialyzed against buffer C (20 mM Tris–HCl, pH 7 50 mM NaCl). The protein was analyzed by SDS-PAGE, using Blue Safe staining (Nzytech). An image of the gel was taken with a Proxima AQ-4 gel documentation system (Isogen) and the resulting amount of protein in the gel bands was quantified using FIJI software  [52].

### Determination of enzyme activity at different conditions of temperature and pH

Activity of purified xylanases was assayed at a range of temperature and pH. The enzyme reactions to be tested at different temperatures were prepared by mixing 180 μL of substrate (1% oat-spelt xylan, in Tris–HCl 50 mM buffer pH 9.0) and 20 μL of purified protein (protein concentration was adjusted to the xylanase assay conditions) and then incubated at 60, 70, 80 or 90 °C, for 10 min. The reaction was stopped by putting the tubes on ice.

Activity as a function of pH was determined using 50 mM buffered solutions, at the following pH values: 5.0 (acetate), 6.0 and 7.0 (phosphate), 8.0, 9.0 and 10.0 (Tris–HCl). The enzyme reactions were prepared by mixing 180 μL of 1% oat-spelt xylan (Sigma) in buffer and 20 μL of purified protein (diluted at a concentration suitable for the DNS assay). The reactions were incubated at 65 °C during 10 min and then stopped on ice.

Production of reducing sugars was determined by adding 100 μL of DNS solution to the reaction tubes that were then boiled for 10 min. Next, 900 μL of miliQ H_2_O was added and the tubes were centrifuged. 300 μL of the supernatant was transferred to 96-well plates and OD_540_ was measured using PowerWave HT equipment, from BioTek Instruments (Winooski, VT, USA).

### Rice straw degradation assays

Rice straw was ground with a coffee grinder. Rice straw powder at 10% (w/v) was suspended in 2% NaOH and heated at 121 °C for 20 min. Aliquots of the suspension were adjusted at different pH (7.0, 9.0 and 10.5) with HCl.

Xylanases activity was assayed using in tubes containing 500 μL of rice straw, at different pH, to which 50 μL of enzyme solution (0.15 units) were added. One unit is defined as 1 μmol of reducing sugars min^−1^ mg^−1^ from oat-spelt xylan, at pH 7.0 and 65 °C. Enzyme treatments were carried out at two temperatures: 65 and 90 °C, during 24 h. After this time, the tubes were centrifuged and 20 μL of 1:10 dilution of the supernatant were used to determine reducing sugars as described above. Analysis of soluble sugars released by the enzymatic treatment was carried out by ion exchange chromatography using a Dionex (Thermo Fisher Scientific) instrument equipped with CarbonPac PA100 column and a pulsed amperometric detector (Dionex Thermo Fisher Scientific). Xylose (Sigma-Aldrich) and xylooligosaccharides, from two to six units (Megazyme) were used as chromatographic standards (Additional file 5: Figure S2).

## Supplementary Information

**Additional file 1: Table S1.** List of domains (PFAM) analyzed in this work.

**Additional file 2: Table S2.** Phylogenetic analysis of the GH11 domain. The figure was generated as described for Figure 1 but including tags for each sequence that shown the accession number and the domain architecture. Sequences tagged with “E” or “A” at the end of the domain architecture correspond to xylanases from eukaryotic organisms or archaea organisms respectively. The meaning of Ct1, Ct2 and Ct3 tags is detailed is the manuscript.

**Additional file 3: Figure S1.** Detailed presentation of the cladogram shown in Figure 1. Information of the accession number, domain architecture and origin of each sequence is provided, as explained for Fig. 1.

**Additional file 4: Table S3.** Synthetic nucleic acid sequences used in this work.

**Additional file 5: Figure S2.** Chromatographic analysis of compounds used as standards: xylose (peak 1) and xylooligosaccharides, xylobiose to xylohexaose (peaks 2 to 6).

## Data Availability

The data sets used and/or analyzed during the current study are available from the corresponding author on reasonable request.

## Abbreviations

GH: Glycoside Hydrolase
DA: Domain Architecture
Xyn: Xylanase
CBM: Carbohydrate-Binding Module
Ct: Carboxy terminal
JTT: Joint transform-domain-translated
PKD: Polycystic Kidney Disease
IPTG: Isopropyl β-d-1-thiogalactopyranoside
OD: Optical Density
PCR: Polymerase Chain Reaction
DNS: Dinitrosalicylic acid
SDS-PAGE: Sodium Dodecyl Sulfate Polyacrylamide Gel Electrophoresis
BSA: Bovine Serum Albumin
