# Supplementary material for: In silico screening and experimental analysis of family GH11 xylanases for applications under conditions of alkaline pH and high temperature
Source: Biotechnol Biofuels. 2020 Dec 7;13:198. doi: 10.1186/s13068-020-01842-5 (PMC7720462; doi:10.1186/s13068-020-01842-5)
Supplement: Supplementary file 2 — Additional file 2: Table S2. Phylogenetic analysis of the GH11 domain. The figure was generated as described for Figure 1 but including tags for each sequence that shown the accession number and the domain architecture. Sequences tagged with “E” or “A” at the end of the domain architecture correspond to xylanases from eukaryotic organisms or archaea organisms respectively. The meaning of Ct1, Ct2 and Ct3 tags is detailed is the manuscript. [file 13068_2020_1842_MOESM2_ESM.pdf]

Name	Accession number	Organism	Sequence
**Xyn1**	AAQ12276.1	*Bacillus halodurans*	AAG***GAGCTC***[SacI]ATGTTCAAGTTTGTAACCAAGGTATTGACGGTTGTCATTGCTGCTACAATTTCCTTTTGCCTGTCTGCTGTCCCAG<br>CTTCAGCTAATACATATTGGCAATATTGGACCGACGGGGGCGGGACCGTCAATGCTACCAACGGACCTGGCGGAAACTACAGTGTGAC<br>TTGGCGTGATACTGGCAATTTCGTTGTAGGCAAAGGGTGGGAGATCGGGAGTCCAAACCGCACTATTCACTATAACGCCGGCGTCTGG<br>GAGCCTAGTGGGAACGGGTATTTGACGCTGTATGGTTGGACTCGTAATCAATTAATTGAATACTACGTGGTGGATAACTGGGGGACAT<br>ATCGTCCAACCGGAACGCACCGTGGCACAGTGGTCTCGGATGGAGGTACTTATGATATTTATACAACCATGCGCTATAATGCGCCTTC<br>TATTGATGGTACACAGACCTTTCAGCAATTTTGGTCGGTCCGCCAGTCTAAGCGTCCTACTGGTAATAACGTCTCAATTACATTCTCT<br>AATCATGTCAACGCGTGGCGTAACGCCGGGATGAACTTAGGAAGCTCATGGTCGTACCAGGTCTTAGCGACAGAGGGTTACCAATCCT<br>CAGGGCGCTCAAATGTGACAGTTTGGTAG***GTCGAC***[SalI]GGC
**Xyn 3**	CAJ87325.1	*Thermobacillus xynylaniticus*	AAG***GAGCTC***[SacI]AACACATATTGGCAGTATTGGACTGACGGGATCGGATACGTTAATGCAACTAACGGCCAAGGAGGGAATTATTCGG<br>TGTCGTGGTCGAACAGTGGAAATTTTGTGATTGGAAAAGGTTGGCAGTACGGTGCGCATAACCGTGTAGTCAATTACAATGCAGGAGC<br>CTGGCAGCCCAACGGCAATGCCTATCTGACGCTGTATGGTTGGACTCGCAACCCCCTGATTGAATATTACGTGGTGGATTCATGGGGT<br>TCATACCGCCCGACAGGGGACTACCGCGGTTCCGTGTATTCGGACGGCGCGTGGTACGACTTGTACCATAGTTGGCGCTACAATGCTC<br>CGTCCATCGACGGGACACAAACCTTCCAGCAGTACTGGTCGGTCCGCCAGCAAAAGCGTCCCACCGGATCTAATGTATCTATCACGTT<br>CGAGAATCATGTAAACGCCTGGGGTGCCGCAGGCATGCCGATGGGCTCGTCGTGGTCATATCAGGTCCTGGCTACAGAGGGTTACTAT<br>AGCAGCGGGTACAGCAATGTCACTGTTTGGTAG***GTCGAC***[SalI]GGC
**Xyn4**	AGA56981.1	*Thermobacillus composti KWC4*	*AAG**GAGCTC**[SacI]ATGGCAGCGGCATTAGCGGCCGTTGCGTTACTTTATGGACTTTTCGCGGTTAATGCCAGCGCTGATCTTAATTGGT*<br>*ACTTTTGGACCAACGGTAACGGCAACGTCAACGCAGTAAATGGCCCAGACGGCCAGTATTCTGTGGATTGGTCAGGGAATGCTCTGTT*<br>*CATTGCAGGAAAGGGATGGCAGATTGGGAGCCCAGGCCGCGTGATCCACTATACCGCCTCAGATTTTCAGACGGATGGAAGCGCCTTC*<br>*TTATTGGTGTACGGATGGATGCGTAACCCGCTGGTGGAATATCGCGTAGTAGAAAATTGGGGGAATTGGCGTCCCGTCACAGCGGATT*<br>*ATCGTGGCAGCGTCTACATTGATGGCGCCTGGTACGACCTTTACCATGCCTTGCGCTTTAATGCCCCAAGCCCAGAGGGAACCCAAAC*<br>*CTTTCAACAGAATTGGTCAGTGCGCCAAACGAAGCGCCCAATCGGTAGCCGCAGTACTGTTACGCTTGAGGCGCACGTAAAGGCTTGG*<br>*GAGGACGCCGGAATGCCGCTTGGTAATAATTGGTTACATCAAATCTTAGCGGTAGAAGGGGGATCTGCGGCAGGTCAAGCCTCCGTGA*<br>*CAGTTTGGTAG**GTCGAC**[SalI]GGC*
**Xyn5**	ACC46361.1	*Dictyoglomus thermophilum*	AAG***GAGCTC***[SacI]TGTTCGATCACATTGACATCGAACGCCAGCGGCACCTTTGACGGGTATTATTACGAGCTTTGGAAGGATACCGGAA<br>ACTGCACTATGACTGTTTATACTCAGGGTCGCTTCTCGTGTCAATGGTCTAATATTAACAACGCGCTTTTCCGCACGGGCAAAAAGTA<br>CAATCAAAATTGGCAGAGTTTAGGGACAATCCGTATTACATATAGTGCCACTTATAATCCAAACGGAAATTCCTATCTGTGCATTTAT<br>GGTTGGTCCACAAATCCTTTGGTTGAATTTTATATCGTAGAATCCTGGGGGAACTGGCGCCCGCCAGGCGCAACCTCGCTGGGGCAAG<br>TGACGATTGATGGGGGTACGTACGACATTTACCGTACAACTCGTGTCAACCAGCCAAGCATTGTTGGGACTGCGACCTTCGATCAGTA<br>CTGGTCAGTACGTACCAGCAAGCGCACATCTGGAACTGTGACAGTAACCGATCACTTCCGCGCGTGGGCAAATCGCGGATTGAATCTG<br>GGGACAATTGACCAGATCACGCTGTGCGTCGAGGGGTACCAGTCTTCAGGGTCAGCTAATATTACACAAAATACCTTCTCTCAAGGGT<br>CGTCATCAGGGAGCAGCGGCGGTTCCAGCGGTAGTACAACTACCACACGCATTGAATGTGAAAACATGTCTCTGAGCGGGCCCTACGT<br>TTCACGTATCACCAACCCGTTTAATGGAATTGCACTGTATGCCAATGGCGATACGGCACGTGCTACGGTTAATTTCCCCGCCTCTCGT<br>AATTATAACTTCCGCTTGCGCGGGTGTGGTAACAATAACAACCTTGCGCGCGTTGACCTGCGTATTGACGGTCGCACGGTGGGTACAT<br>TCTATTACCAGGGCACATATCCATGGGAGGCTCCGATTGATAATGTTTACGTATCCGCAGGGTCACATACTGTCGAGATCACTGTCAC<br>TGCTGACAACGGCACGTGGGATGTTTATGCAGACTATTTGGTAATCCAGTAG***GTCGAC***[SalI]GGC
**Xyn6_F1**	ACV12129.1	*Halorhabdus tiamateaSARL4B*	AAG***GAGCTC***[SacI]ATGGGGCGCCATAATATTGAGGACGCCGCTGAAGAGAGCAGTGACAACGATCGTTTAGGCCAATTTGACCGCCGTT<br>CCTACCTGAAGGGTGCGGCCGCTACAGTGGCAACGGGCTTAGGAGTTGGGAGTATTGCTTCTCCGGCGGCAGCTATTACAGAAAACCA<br>GACAGGGACACACGACGGATATTTCTACAGTTTCTGGACCAATGATCAAGGCAGTGTCGAGATGACTTTGGAAAGCGGGGGTTCTTAT<br>TCGGTTGATTGGTCTGATACTGGAAATTTCGTATGCGGGAAGGGATGGCAAACAGGTTCGGACCGTGATATCGAGTATACGGCGAACT<br>ACAATCCGCAAGGGAATTCGTACCTGTGCTTGTACGGTTGGACAACAGACCCCCTTGTGGAATATTACATTATTGAAGATTATGGAAG<br>CTATAAGCCTGGTGATCAATCTCAGGGCACACATACCACAGACGGGTCTACTTATGAAATGTACACTTCAGAACGTGTTGAGAAACCT<br>TCAATTGAGGGCACAGCGACTTTTACCCAGTACTGGTCAATCCGCCAAAACTCGCGCACCAGTGGAACCATCACAACGAGTAATCATT<br>TTGACGCCTGGGAATCTGCAGGTCTGAATATGGGTTCTCACGACTATCAAATTCTGGCCACGGAGGGCTATCAGTCTTCTGGCAGCAG<br>CAGTGTCACCGTCGGATCTAGTGGAGGTGGGGGTGGCGGCGGTGGGGGAGGGGGCGGTGGTGGTGGGGGTGGCTCCGGATCACAACAA<br>CCTTACAAC***GGTACC***[KpnI]GGC
**Xyn6_F2**	ACV12130.1	*Halorhabdustia mateaSARL4B*	GGC***GGTACC***[KpnI]CCTCACTCAATTCCCGGCACAATTCCGGCGGAGGAATACGACCAGGGTGGGAGTGGGGTCGCATACTCGGACAATA<br>CCTCAGAGAATGAGGGGGGCGCAATGCGCACCGGTGAGGGTGTTGACATTTCCTCGAACAGTGCGGGTAGTGGCTACTCAATTGGGTA<br>TATCGAGAGTGGCGAGTGGGTGGAATATACGGTAGACGTTCAACAGAGCGGGGATTACACCTTAGACGCTTTGGTGGCTTCCGATTCT<br>GGTGGAGGGTCCTTCCATTTAGAGGTCAACGGACAGAACGTATCCGGTAACGTAAGTTTTGGCGCAACCGGAGGGTGGGACTCTTGGG<br>AGACCGTCAGCACGTCTGGGGTGAGTTTGGATGCTGGCCAGCAAGTGATCCGTGTGAGCATGGATGAATCATGGTGGGACTTAAACAC<br>CTTCGACCTTTCCTTGGATGGGGGTGGGGGCGGCGGTGGTGGCGGCGGAGGGGGTGGCGGAAACGGCGGAACTGGAGGGGGGGGTGGT<br>ACATCCGGTGACTTGGTAGCAGAAATTGACCCAAACACCACCAGCGCGTCTACTGGGGATCTGGTCCAGTTTAATATCAATGATACGA<br>CCGGGTCAGGAAACTATATTTCTTCACTTGAATGGGACTTGGGAAATGGTGTGACCGGATCAGGGTGGTATATTGATGAACGTTATCA<br>GTCTGTTGGTTCTTATACAGTGACTCTTACAGCTACGGATAATGAGGGGCGTTCTACTACTGATGAGGTTACTGTAACGATCTCGTAG<br>***GTCGAC***[SalI]GGC
**Xyn7**	ACV12130.1	*Halorhabdustia mateaSARL4B*	AAG***GAGCTC***[SacI]ATGGGTCGCCATAATATTGAGGACGCCGCGGACGAAAGCAGCGACAATGGGCGCATTGGACGTTTCGACCGCCGCA<br>GTTACTTGAAGGGGGCTGCCGCCACAGTAGCAACTGGACTTGGTATTGGATCAATTGCTTCACCGGCGGCCGCTATCACATCGAATCA<br>GACTGGGAATGACTCAGGATATTATTACTCGTTCTGGACCAACGCTGAGGGCACGGTCGAGATGACGTTGGGTGACGGAGGAAACTAC<br>AGCGTGGACTGGAGTGATACAGGAAATTTCGTATGTGGTAAGGGATGGCAAACCGGGGGTCGTCGTGATGTGGACTATACCGCGAATT<br>ATAATCCGTCGGGCAATTCGTACTTGTGCCTGTATGGCTGGACTACAGACCCCTTGGTAGAATATTACATCATCGAAGATTACGGTAG<br>CTACAAGCCAGGCGACTCTTACCAGGGTACGCATACTACAAATGGGTCTACTTATGAGATCTATACCAGTGAACGCGTGAACAAACCA<br>AGCATTGAGGGTAATGCTACCTTCACCCAATACTGGTCAGTCCGCCAGAATAGCCGCACATCAGGTACCATCACTACCGGAAACCATT<br>TCGATGCATGGGAGAACCATGGGCTGCCTATGGGAAATCACGATTACATGATCTTAGCCACAGAGGGCTATCAAAGCTCTGGCTCCTC<br>TGAGGTTTGGGTTGGTGCCGATGGGTCAGGAGGTAGTGGTGGGGGTGGCGGAGGGGGAGGGGGCGGTGGTGGCTCGGGGGGTGGTGGA<br>GGGGGTTCCTCCGGTAGTTCGAATCCTCCGGCGTCAGGGACCTACTCCATTCAGAACGTAAATTCAGGCAAGGGGATGGATGTGGCTG<br>GCCAAAGCACAAGCGACGGCGCCAATGTACAGCAGTATTCATACTGGGGTGGTGAAAATCAGCAATGGAACGTCGAAGATACGGGCGG<br>CGGGCAGTTTCGCATTCAGAATGTCAATAGTGGTAAAGTCTTAGATGTCGCGAACCAGAGCACGGAAGATGGTGCAAACGTTCACCAG<br>TACTCGGACGGTGGAGGCGACAATCAGCGCTTTTACCTTAATGACCAGGGAGGCGGGCAGTACCATATTCAACCGGTCCATTCGCAAA<br>AAGCTGTAGAGATTACATCGAGTTCGACCTCGGATGGGGCTAATGTGCAGCAGTACGACTGGCACGGCGGAAATAACCAACTGTGGAC<br>CTTTGAGAGTGTGTAG***GTCGAC***[SalI]GGC
**Xyn8**	AAA21480.1	*Thermobifida fusca*	AAG***GAGCTC***[SacI]GCAGTGACTTCTAACGAAACAGGATATCATGATGGGTACTTCTATTCATTCTGGACCGACGCACCGGGGACTGTTA<br>GTATGGAGCTGGGTCCGGGTGGTAACTATTCAACCTCGTGGCGTAACACCGGCAACTTCGTCGCGGGCAAAGGGTGGGCTACGGGTGG<br>TCGCCGCACAGTTACTTATTCGGCCAGTTTTAATCCTTCGGGAAATGCGTATCTTACGCTTTATGGCTGGACACGTAACCCCTTGGTT<br>GAATATTACATCGTGGAGAGCTGGGGGACGTACCGTCCCACGGGCACATATATGGGAACCGTCACCACAGATGGTGGCACGTACGATA<br>TCTATAAAACTACCCGCTACAACGCCCCGAGTATCGAGGGTACTCGTACCTTCGACCAGTACTGGTCAGTTCGCCAAAGCAAACGTAC<br>GAGCGGAACCATTACCGCTGGTAACCACTTTGATGCTTGGGCTCGTCATGGGATGCATCTGGGTACGCACGACTATATGATTATGGCC<br>ACGGAAGGTTATCAAAGCAGCGGAAGTAGCAATGTCACACTTGGAACATCGGGAGGAGGTAACCCCGGTGGCGGAAACCCACCCGGAG<br>GTGGGAATCCTCCCGGTGGAGGCGGTTGTACAGCTACTTTAAGTGCTGGACAGCAGTGGAATGACCGTTACAACTTAAATGTGAACGT<br>CTCTGGTAGCAACAACTGGACTGTGACCGTCAACGTACCATGGCCAGCACGCATCATCGCCACCTGGAACATCCACGCCTCGTATCCC<br>GACTCTCAGACACTTGTGGCCCGTCCAAACGGTAACGGAAACAATTGGGGGATGACAATCATGCACAACGGTAATTGGACCTGGCCGA<br>CCGTATCCTGCAGCGCCAATTAG***GTCGAC***[SalI]GGC
