# Supplementary figures and images for: In silico screening and experimental analysis of family GH11 xylanases for applications under conditions of alkaline pH and high temperature
Source: Biotechnol Biofuels. 2020 Dec 7;13:198. doi: 10.1186/s13068-020-01842-5 (PMC7720462; doi:10.1186/s13068-020-01842-5)

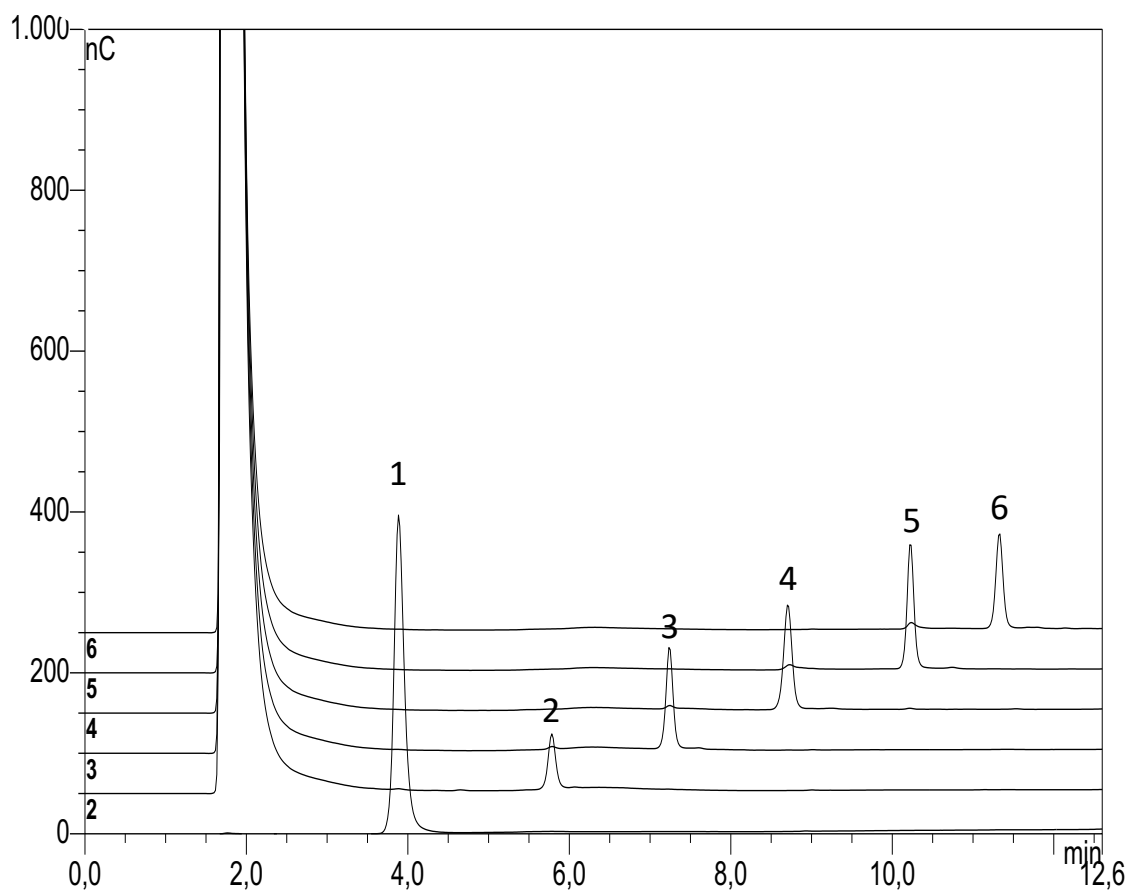

Supplement: Supplementary file 3 — Additional file 3: Figure S1. Detailed presentation of the cladogram shown in Figure 1. Information of the accession number, domain architecture and origin of each sequence is provided, as explained for Fig. 1. [file 13068_2020_1842_MOESM3_ESM.pdf]
