# Supplementary material for: In silico screening and experimental analysis of family GH11 xylanases for applications under conditions of alkaline pH and high temperature
Source: Biotechnol Biofuels. 2020 Dec 7;13:198. doi: 10.1186/s13068-020-01842-5 (PMC7720462; doi:10.1186/s13068-020-01842-5)
Supplement: Supplementary file 5 — Additional file 5: Figure S2. Chromatographic analysis of compounds used as standards: xylose (peak 1) and xylooligosaccharides, xylobiose to xylohexaose (peaks 2 to 6). [file 13068_2020_1842_MOESM5_ESM.docx]

| PFAM name | PFAM code | Code in this work |
| --- | --- | --- |
| Dockerin_1 | PF00404.17 | Dockerin_1 |
| Malectin_like | PF12819.6 | Malectin_like |
| Lipase_GDSL_2 | PF13472.5 | Lipase_GDSL_2 |
| CBM9_1 | PF06452.10 | CBM9 |
| Cellulase | PF00150.17 | Cellulase |
| He_PIG | PF05345.11 | He_PIG |
| RicinB_lectin_2 | PF14200.5 | Ricin_B_lectin |
| Ricin_B_lectin | PF00652.21 | Ricin_B_lectin |
| Esterase | PF00756.19 | Esterase |
| CBM_6 | PF03422.14 | CBM6 |
| Flg_new | PF09479.9 | Flg_new |
| CBM_2 | PF00553.18 | CBM2 |
| Glyco_hydro_11 | PF00457.16 | GH11 |
| Glyco_hydro_10 | PF00331.19 | GH10 |
| Glyco_hydro_16 | PF00722.20 | GH16 |
| Malectin | PF11721.7 | Malectin |
| PKD | PF00801.19 | PKD |
| SASA | PF03629.17 | SASA |
| CBM_5_12_2 | PF14600.5 | CBM5-12_2 |
| CBM_10 | PF02013.15 | CBM10 |
| Lipase_GDSL | PF00657.21 | Lipase_GDSL |
| CBM60 | PF16841.4 | CBM60 |
| Polysacc_deac_1 | PF01522.20 | Polysacc_deac_1 |
| CBM_4_9 | PF02018.16 | CBM4-9 |
| CBM_1 | PF00734.17 | CBM1 |
| CBM_10 | PF02013.15 | CBM10 |
| Glyco_hydro_62 | PF03664.12 | GH62 |
