# Supplementary material for: In silico screening and experimental analysis of family GH11 xylanases for applications under conditions of alkaline pH and high temperature
Source: Biotechnol Biofuels. 2020 Dec 7;13:198. doi: 10.1186/s13068-020-01842-5 (PMC7720462; doi:10.1186/s13068-020-01842-5)

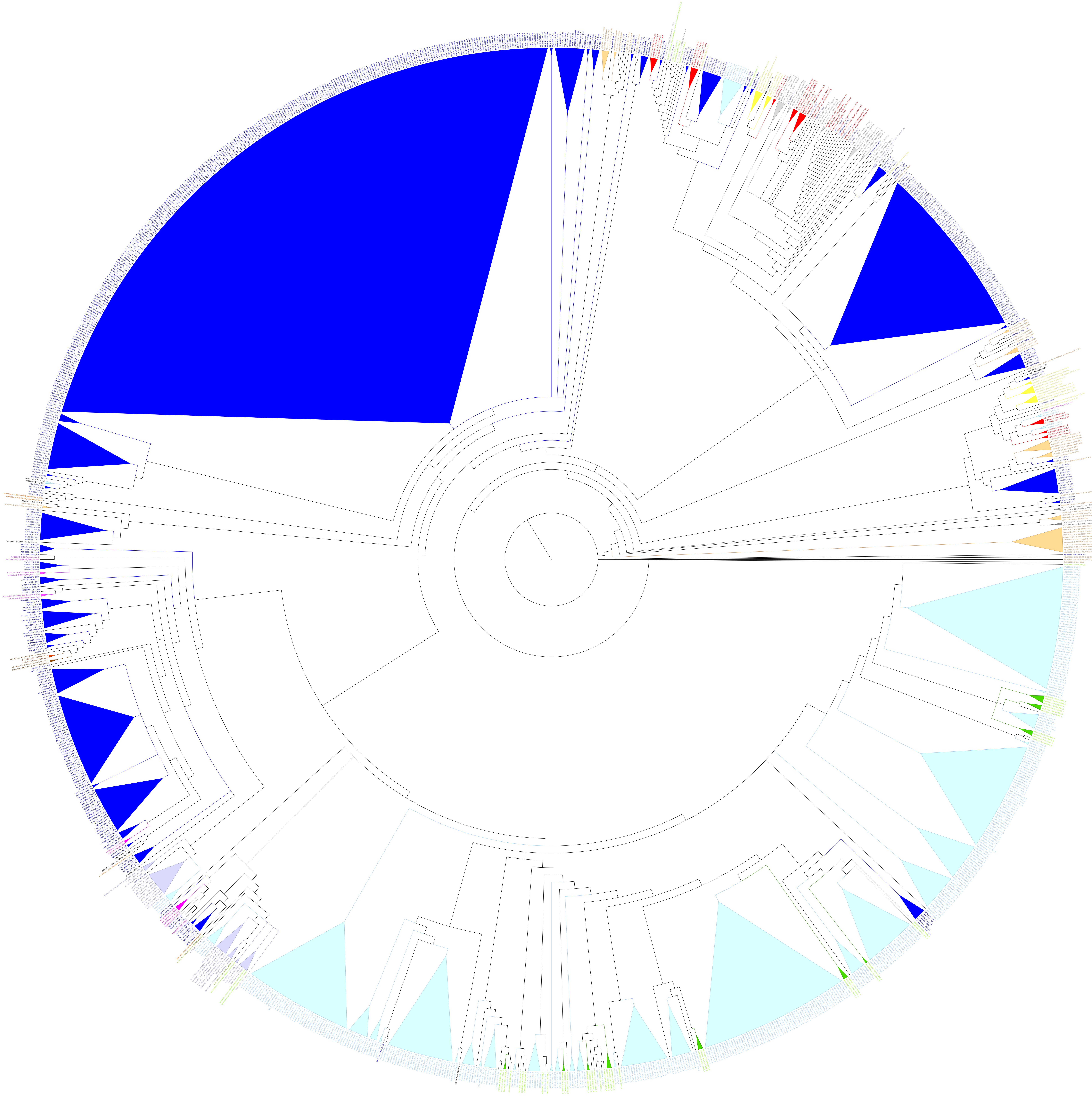

Supplement: Supplementary file 4 — Additional file 4: Table S3. Synthetic nucleic acid sequences used in this work. [file 13068_2020_1842_MOESM4_ESM.pdf]
